# Supplementary material for: Development of Fenofibrate/Randomly Methylated β-Cyclodextrin-Loaded Eudragit® RL 100 Nanoparticles for Ocular Delivery
Source: Molecules. 2022 Jul 25;27(15):4755. doi: 10.3390/molecules27154755 (PMC9370055; doi:10.3390/molecules27154755)
Supplement: Supplementary file 1 [file molecules-27-04755-s001.zip › molecules-1792390-supplementary.pdf]

Supplementary Materials

Development of Fenofibrate/Randomly Methylated β-Cyclodextrin–Loaded Eudragit® RL 100 Nanoparticles for Ocular Delivery

Soe Yadanar Khin [1], Hay Man Saung Hnin Soe [1], Chaisak Chansriniyom [1], Natapol Pornputtapong [1], Rathapon Asasutjarit [2], Thorsteinn Loftsson [3] and Phatsawee Jansook [1,*]

[1] Faculty of Pharmaceutical Sciences, Chulalongkorn University, 254 Phyathai Road, Pathumwan, Bangkok 10330, Thailand; soeyadanarkhin1993@gmail.com (S.Y.K.); haymansaunghninsoe@gmail.com (H.M.S.H.S.); Chaisak.Ch@chula.ac.th (C.C.); natapol.p@chula.ac.th (N.P.)

[2] Faculty of Pharmacy, Thammasat University, 99 Moo 18 Paholyothin Road, Klong Luang, Rangsit, Pathum Thani 12120, Thailand; rathapon@tu.ac.th

[3] Faculty of Pharmaceutical Sciences, University of Iceland, Hofsvallagata 53, IS-107 Reykjavik, Iceland; thorstlo@hi.is (T.L.)

*Correspondence: phatsawee.j@chula.ac.th; Tel.: +662-218-8273

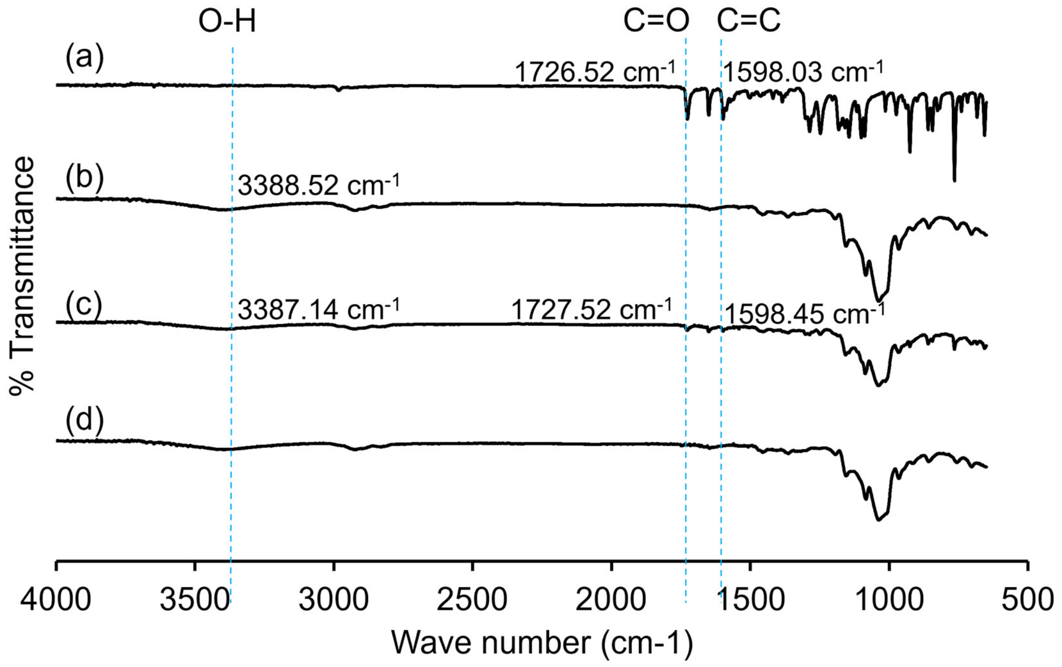

Figure S1. The FTIR spectra of (a) pure FE, (b) pure RMβCD, (c) PM FE/RMβCD and (d) FD FE/RMβCD

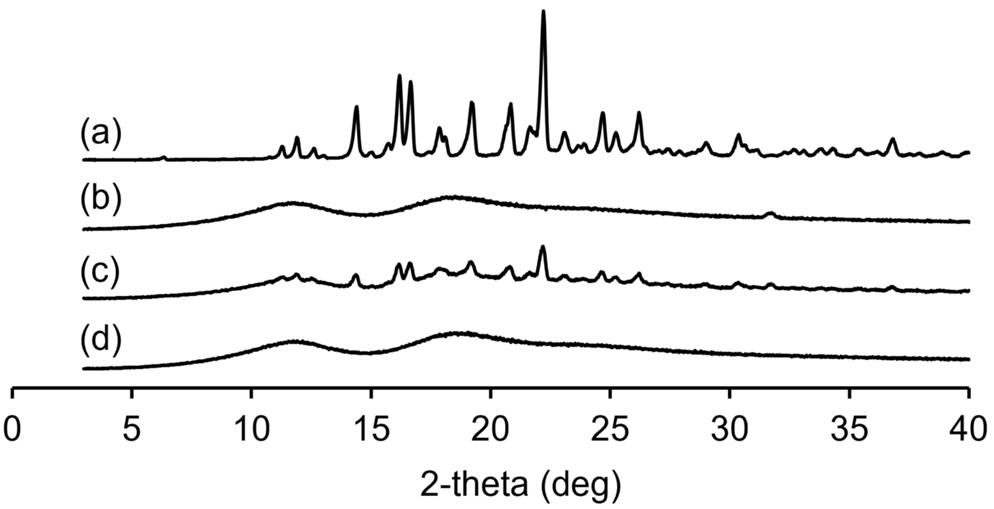

Figure S2. The PXRD spectra of (a) pure FE, (b) pure RMβCD, (c) PM FE/RMβCD and (d) FD FE/RMβCD

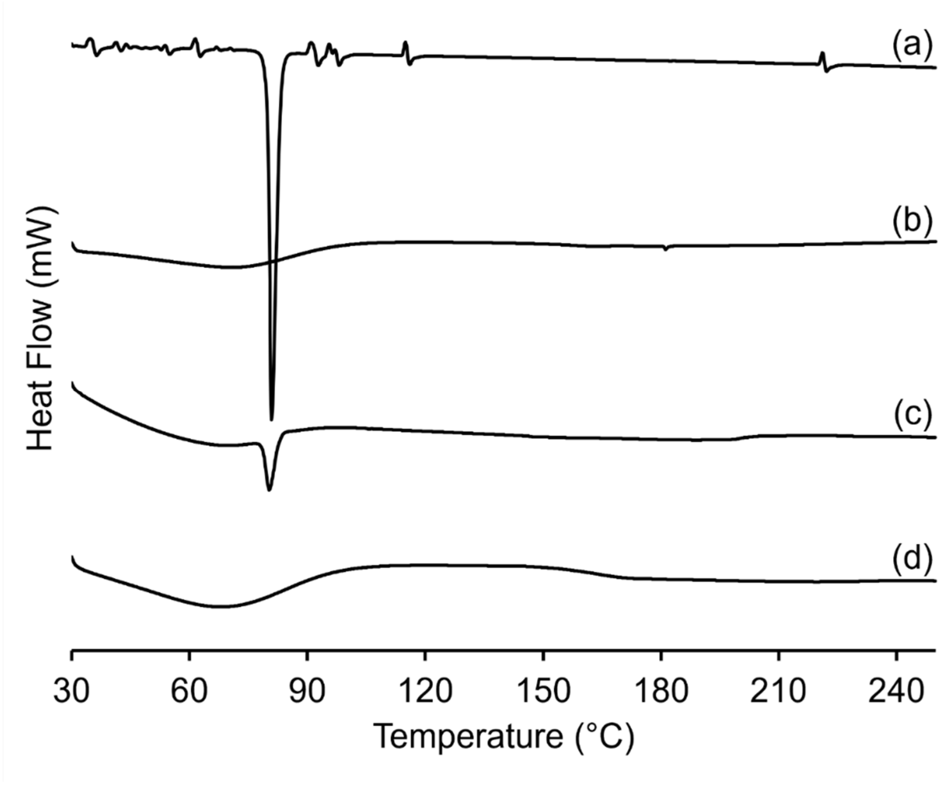

Figure S3. The DSC thermograms of (a) pure FE, (b) pure RMβCD, (c) PM FE/RMβCD and (d) FD FE/RMβCD

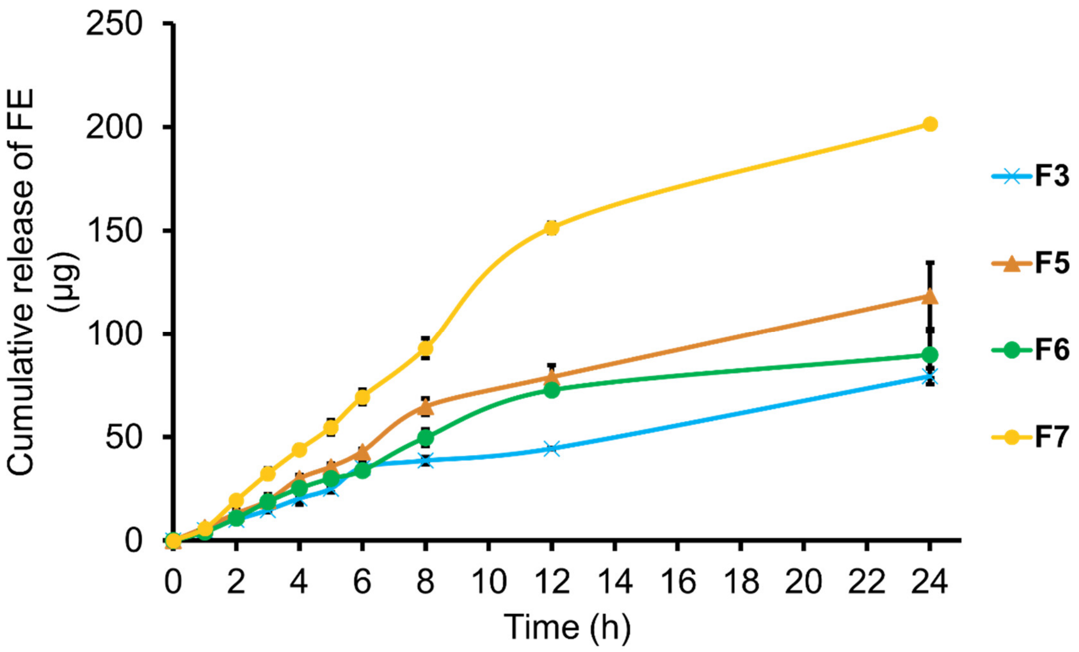

Figure S4. In vitro cumulative release profiles of FE in FE loaded Eudragit® RL nanoparticles formulations

Table S1. Kinetic release rate constants (K), correlation coefficient (R^2) and diffusion exponent (n) of various models (*n*=3)

**Formulations**	**Kinetic models**								
	Zero order		First order		Higuchi		Korsmeyer-Peppas		
	K_0	R^2	K_1	R^2	K_h	R^2	n	K	R^2
F3	0.220	0.953	-0.001	0.956	1.156	0.952	0.863	0.397	0.977
F5	0.235	0.953	-0.001	0.956	1.226	0.944	0.942	0.464	0.976
F6	0.265	0.899	-0.001	0.903	1.421	0.937	0.984	0.419	0.959
F7	0.295	0.941	-0.001	0.945	1.537	0.927	1.119	0.559	0.963
